# Conformal Curved-Electrode Sensor with High-Frequency Optimization for Distributed Conductivity Monitoring in Shipboard Desalination Pipelines

**DOI:** 10.3390/s25175464

**Published:** 2025-09-03

**Authors:** Wenlong Wang, Junya Shi, Cong Chen, Haibin Yang, Kai Li, Zhiying Zheng, Linzhou Huang

**Affiliations:** 1 Department of Basic Courses, Naval University of Engineering, Wuhan 430033, China; 1920191008@nue.edu.cn (W.W.); cckx7145@163.com (C.C.); 13477058881@139.com (H.Y.); likai16@mails.ucas.ac.cn (K.L.); 2 School of Computer Science, Wuhan Donghu College, Wuhan 430212, China; 3 School of Electrical Engineering, Naval University of Engineering, Wuhan 430033, China; m24180802@nue.edu.cn; 4 Institute of Noise and Vibration, Naval University of Engineering, Wuhan 430033, China; 13577434056@163.com

**Keywords:** conductivity sensor, online pipeline monitoring, curved electrodes, seawater desalination, measurement device

## Abstract

Current seawater desalination systems on ships face several limitations including outdated concentration detection methods, low detection accuracy, and insufficient real-time monitoring capabilities. This study addresses these issues by developing a concentration measurement device based on two-electrode conductivity measurement principles. The key innovation involves transforming conventional parallel plates into curved electrode plates that can be embedded directly into pipelines, enabling real-time concentration monitoring in shipboard seawater desalination systems. We established an equivalent circuit model and conducted simulation analysis of amplitude–frequency and phase–frequency response characteristics to guide excitation signal frequency selection. Using 3D printing technology, we fabricated pipeline components and manually processed curved electrode plates, then assembled experimental devices and determined optimal working parameters through systematic measurements of solution conductivity versus frequency and concentration. Laboratory testing with known concentration saline solutions demonstrated high measurement accuracy, with the device achieving a relative error of only 1.457% for 3.5% NaCl solution (simulated seawater) and 3.000% for commercial saline (0.9% NaCl) after calibration. Finally, we integrated a PLC control system for automated concentration measurement and display. Compared to traditional devices that require sampling in static water environments, this system can be distributed throughout shipboard desalination systems, providing more convenient, accurate, and efficient monitoring capabilities.

## 1. Introduction

Seawater desalination is important for addressing global water scarcity [1], especially for long-distance vessels and mobile platforms where ship-borne seawater desalination systems are essential equipment. While many countries have invested in research and development for miniaturization, integration, and intelligence of seawater desalination devices, online monitoring technology remains a significant gap in system operation.

Current ship-borne seawater desalination systems usually only test at inlet and outlet points, lacking online monitoring within pipeline sections. When desalination efficiency decreases, entire filter elements often need replacement, causing high maintenance costs and low operational efficiency. Therefore, developing real-time, distributed online monitoring technology has become an industry priority.

Conductivity measurement to reflect salt content is a well-established method in water quality monitoring [2,3]. Current conductivity measurement technologies mainly use electromagnetic or electrode-type micro-probe measurement approaches. These instruments work well in static environments and regular rectangular containers. However, considering the high flow rates of seawater in desalination system transmission pipelines, high impurity content, significant ship vibration [4], harsh measurement environments, and limited ship space, the limitations of such devices become increasingly apparent.

Conventional electrode-type sensors predominantly rely on a parallel-plate configuration [X, Y]. Despite their widespread use, this fundamental geometry suffers from several inherent limitations that are exacerbated in challenging pipeline environments.

Electrode Polarization: In low-frequency measurements, electrode polarization effects caused by the double-layer at the electrode–solution interface significantly increase impedance and distort readings, especially in high-conductivity media like seawater [2]. While four-electrode systems are explicitly designed to overcome this issue by using separate pairs for current injection and voltage sensing [5], they introduce increased complexity, cost, and physical size. For this study, which prioritizes simple, low-cost, and robust integration into pipeline systems, a two-electrode configuration was selected. The polarization effect is effectively mitigated by employing optimized high-frequency AC excitation and chemically stable, inert electrode materials [2], striking a balance between performance and practical applicability for industrial in situ monitoring.

Non-Uniform Field Distribution: The assumption of a uniform electric field between parallel plates is invalidated by fringe effects, leading to field distortion particularly near electrode edges [6]. This results in non-uniform current density and measurement errors that are difficult to compensate for.

Integration and Practical Challenges: Crucially, the rigid, planar structure of parallel plates is fundamentally incompatible with cylindrical pipes. This creates inevitable air gaps (d_g) when mounted, which drastically distort the electric field, introduce signal instability, and are a primary source of measurement error [Z]. Furthermore, this non-conformal design makes seamless integration into pipelines impossible, often requiring external flow cells that complicate the system.

While research has focused on addressing some issues, like polarization, through advanced electronics or compensation algorithms [7,8,9], the core geometric and integration limitations remain unaddressed. Therefore, a fundamental redesign of the sensor geometry itself is necessary.

To address these limitations, we developed an online concentration measurement device based on two-electrode conductivity measurement principles. The key innovation is transforming conventional parallel plates into curved electrode plates that can be embedded directly into pipelines, enabling real-time monitoring of seawater concentration in shipboard desalination systems. This conformal design eliminates the critical air gap issue, ensures a stable electrode–pipe interface, and promotes a more representative field distribution within the flowing medium.

## 2. Measurement Principle

According to physical chemistry principles [10], when strong electrolyte solutions ionize in liquid and conduct electricity, they follow Faraday’s law: the number of ionized ions is proportional to the circuit current. When the voltage across solution electrodes stays constant, and the solution shape and volume remain unchanged, the equivalent conductivity of the solution is proportional to the number of ionized ions.

The basic principle of conductivity measurement for saline concentration [9,11,12] states that under certain temperature and concentration ranges, higher salt solution concentration results in more charge carriers and stronger conductivity. The equivalent conductivity of the conductivity cell can be used to characterize its conductive ability, establishing the relationship between solution equivalent conductivity and solution concentration. In dilute saline solutions (mass fraction < 5%), solution conductivity is less affected by temperature, ions can be completely ionized, and solution conductivity is proportional to solution concentration.

The two-electrode conductivity method for measuring saline concentration [13,14] uses two conductive electrode plates to apply current in the conductive saline solution between the plates, measures the voltage between the plates to obtain the conductivity of the solution, fits the relationship curve between conductivity and solution salt content, and then quickly determines the concentration of any unknown solution under the same measurement conditions. This method has established applications in industrial settings [14].

## 3. Equivalent Circuit

**Figure 1:**
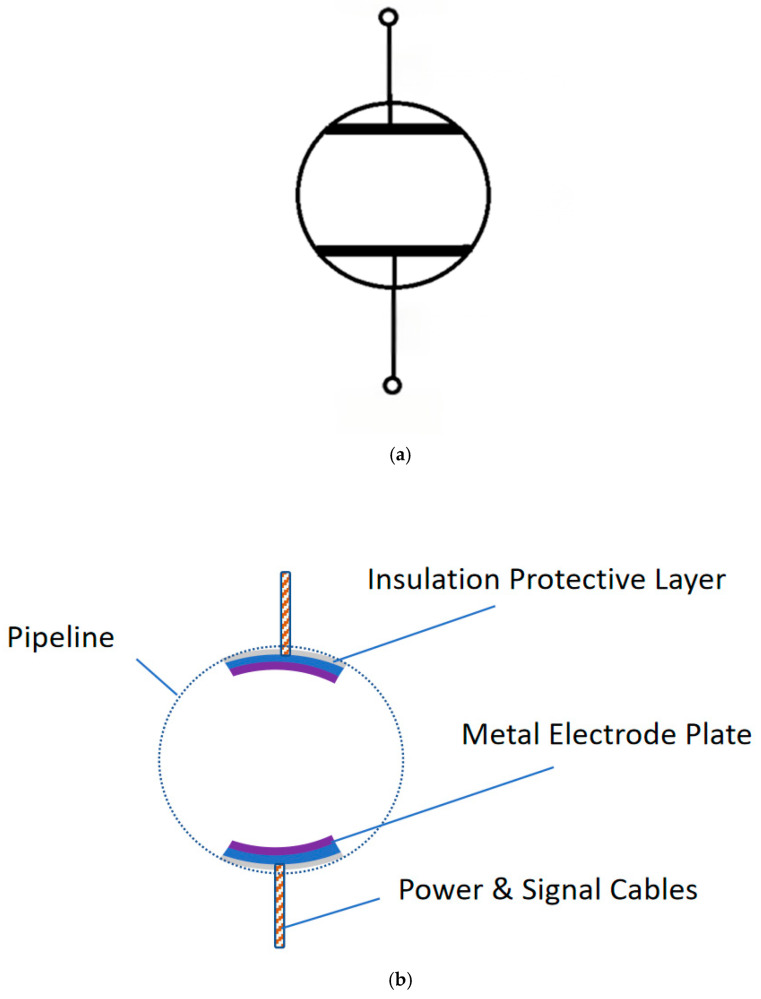
(**a**) Schematic diagram of the conventional parallel-plate electrode. (**b**) Schematic of the in-pipeline seawater conductivity measurement device and its curved electrodes.

The measurement device designed in this project has a structural diagram as shown in Figure 1.

In Figure 1, two metal electrode plates are designed as cylindrical surfaces to fit the inner wall of the pipeline, while their back sides are equipped with insulating protective layers to ensure that current flows only on one side of the electrode plates, reducing the impact of electrode polarization reactions on measurement results. The conductivity measurement principle of this device follows the same approach as two-electrode conductivity measurement with conventional flat electrode plates, and similar equivalent circuits are used for analysis below.

To elucidate the fundamental innovation of our sensor design, a comparative schematic of the conventional parallel-plate electrode configuration versus our proposed conformal curved-electrode is presented in Figure 1. The conventional design (Figure 1a), when mounted on a cylindrical pipe, suffers from two critical limitations: (1) the formation of air gaps (d_g) due to its rigid, flat structure failing to conform to the curved pipe wall, which distorts the electric field and introduces measurement errors; and (2) a restricted sensing region where the electric field is concentrated, preventing representative measurement across the entire pipe cross-section. In contrast, our flexible curved-electrode design (Figure 1b) is engineered to perfectly conform to the pipe’s inner curvature, eliminating air gaps (d_g ≈ 0) and creating a stable electrode–medium interface. More importantly, it facilitates a distributed and uniform electric field that permeates a larger volume of the medium, enabling a more accurate and representative measurement of bulk fluid properties. This direct comparison highlights the key structural advantages that underpin the enhanced performance of our proposed sensor.

**Figure 2:**
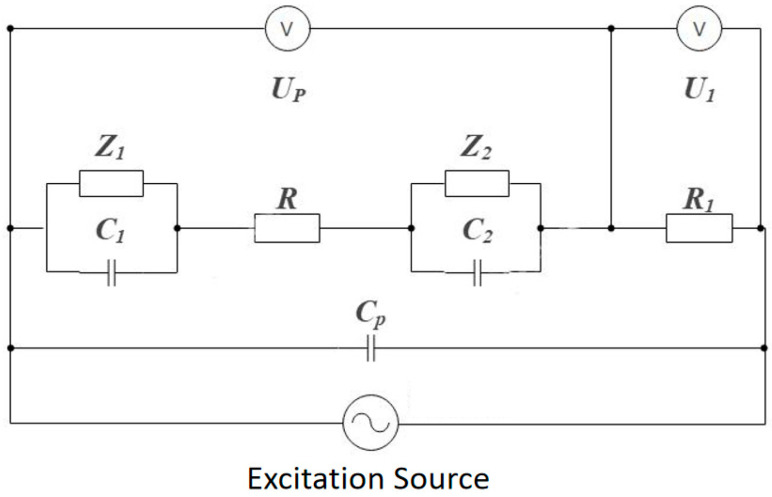
Equivalent circuit of two-electrode measurement device.

During measurement, an external power source applies excitation signals to the two electrode plates, generating large instantaneous currents (reaching more than 10 mA) on the electrode surface. Due to electrochemical reactions [3], electrode polarization and double-layer effects occur on the electrode surface; meanwhile, the distributed capacitance of the conductivity cell wires also affects the circuit. Considering all these factors, the equivalent circuit diagram of the measurement device can be represented as shown in Figure 2.

Where $C_{1}$ and $Z_{1}$, $C_{2}$ and $Z_{2}$ are the equivalent impedances affected by electrode polarization and double-layer effects during excitation electrode reactions, $C_{p}$ is the distributed capacitance of the wires, R is the resistance of the solution to be measured, and $R_{1}$ is the voltage divider resistance of the test circuit. $U_{1}$, $U_{P}$, and U are the voltage across the voltage divider resistance of the test circuit, the voltage across the two electrode plates, and the voltage across the solution to be measured, respectively. According to the circuit diagram, we can obtain the equivalent impedance $R_{P}$ between the electrodes as follows:

(1) $$R_{p}=\frac{\frac{-Z\;j}{\omega \;C_{1}}}{Z_{1}-\frac{1}{\omega \;C_{1}}j}\;+\;R\;+\;\frac{\frac{-Z\;j}{\omega \;C_{2}}}{Z_{2}-\frac{1}{\omega \;C_{2}}j}$$

Since the two electrode plates are identical, their spatial positions have symmetry, and AC current is used, it can be considered that $Z_{1}=Z_{2}=Z$ and $C_{1}=C_{2}=C$. Equation (1) can be further simplified as follows:

(2) $$R_{p}=\frac{2Z}{{\left(Z\omega C\right)}^{2}\;+\;1}\;-\;j\;\frac{2Z^{2}\omega C}{{\left(Z\omega C\right)}^{2}\;+\;1}\;\;+\;\;R$$

Further considering the parallel connection of $R_{P}$ and $C_{P}$, then in series with $R_{1}$, from Equation (2) we find the following:

(3) $$\frac{U_{P}}{U_{1}}=\frac{R_{P}/\left(1\;+\;j\omega C_{P}R_{P}\right)}{R_{1}}$$

Since electrochemical reaction processes cannot be directly measured, there are unknown parameters Z, C, etc., in the actual test circuit that vary with experimental conditions. Therefore, in practical applications, working conditions should be appropriately designed based on the characteristics of the equivalent circuit, such as selecting working frequency bands and external excitation current magnitude, to avoid the impact of these unknown parameters on measurement results and ensure stable and reliable test results.

Further review of the literature shows that [10,11], when applying AC signals to the measurement device, the path formed by $C_{1}$, $C_{2}$, and R is the main channel for AC current. When the applied voltage is not high enough to cause the Faraday effect on $Z_{1}$ and $Z_{2}$, this channel conducts with small impedance, so the impedance of the entire conductivity cell mainly depends on R; and, generally, the values of $C_{1}$ and $C_{2}$ are much larger than $C_{p}$ ($C_{1}$, $C_{2}$ are at the μF level while $C_{p}$ is at the pF level). Under high-frequency excitation, the capacitive reactance of $C_{1}$ and $C_{2}$ is very small and can be ignored. Therefore, under high-frequency and low-voltage signal excitation, the above equivalent circuit can be further simplified.

Considering the magnitude of $C_{p}$, the circuit can be further simplified under certain conditions. For example, when using MHz-level signals for excitation, $C_{p}$ can be calculated to be around $10^{4}\;\Omega$, which is much larger than the impedance of the conductive solution and can be ignored in the circuit. Therefore, in practical applications, by selecting excitation signal frequency bands that meet the conditions, it can also be considered that the current through the constant resistance $R_{1}$ equals the current through R, at the following time:

(4) U=UP, R=ReZp=UU1R1

Through the above principle analysis, it can be seen that appropriate selection of excitation signal frequency can simplify the test principle, simplify the processing of test data and result analysis, and ensure test accuracy. Therefore, the selection of excitation signal frequency bands is a very important aspect in the device design process of this project.

### 3.1. Simulation Analysis of Amplitude–Frequency and Phase–Frequency Characteristics of Equivalent Circuit

Due to unknown parameters Z, C, etc., in the equivalent circuit that vary with experimental conditions, in experimental tests, the impedance of the solution to be measured is actually calculated using $U_{P}$, $U_{1}$, and $R_{1}$. To understand the impact of excitation signal frequency on test results, we use Matlab (2022b) to simulate and analyze the amplitude–frequency and phase–frequency characteristics of the impedance across the electrode plates in the equivalent circuit based on Figure 2 and Equation (3).

The calculation method is as follows:

(5) $$\frac{U_{P}}{U_{1}}=\frac{R_{P}/\left(1+j\omega C_{P}R_{P}\right)}{R_{1}}=\left|\frac{U_{P}}{U_{1}}\right|e^{j\Delta \Phi }$$

Equation (5) is the calculation formula for simulation analysis.

**Figure 3:**
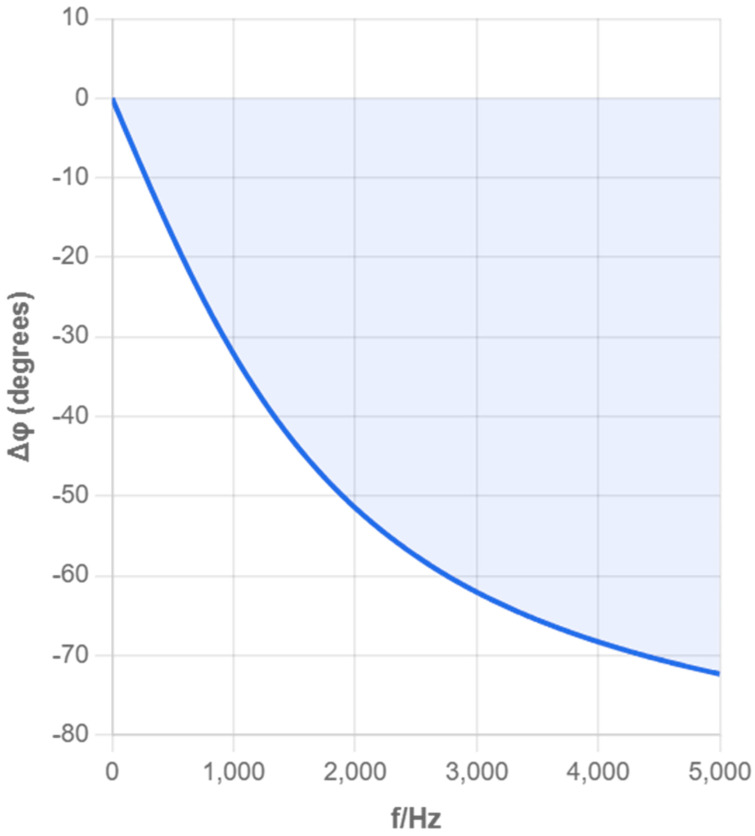
Phase–Frequency Response Curve (0–5 kHz).

**Figure 4:**
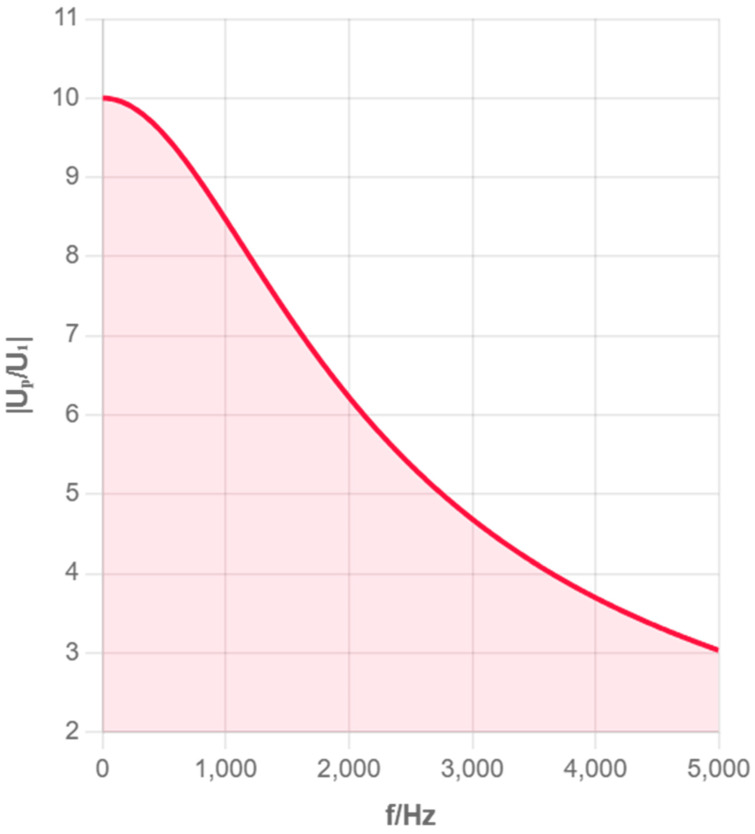
Amplitude–Frequency Characteristic (0–5 kHz).

**Figure 5:**
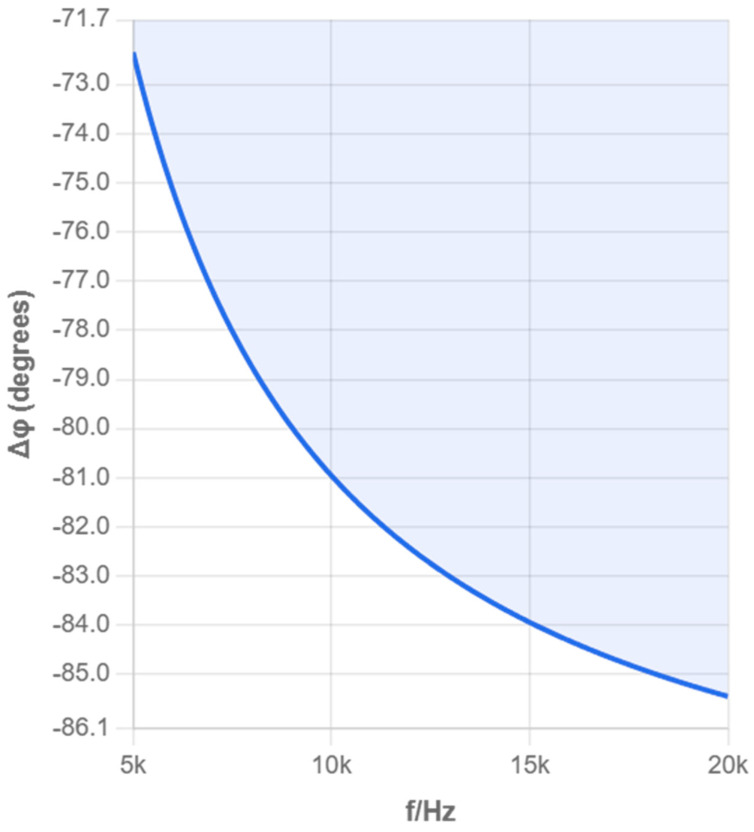
Phase–Frequency Characteristic.

**Figure 6:**
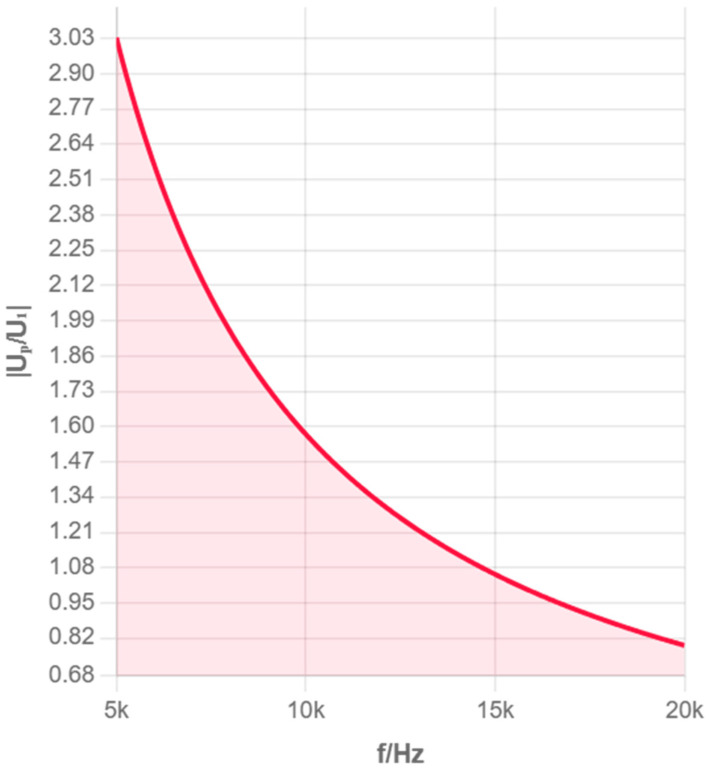
Amplitude–Frequency Characteristic (5–20 kHz).

According to the parameter values of distributed capacitance and other parameters in the references [12,15,16], taking $C_{1}=60\;\mu$F, $C_{p}=10\;$F, R=100 Ω, Z=500 MΩ, we, respectively, plot the amplitude–frequency and phase–frequency characteristics of the impedance of the solution to be measured in the ranges of 0–5 kHz and 5–20 kHz, i.e., the $\frac{U_{P}}{U_{1}}∼f$ and ΔΦ∼f curves, as shown in Figure 3, Figure 4, Figure 5 and Figure 6, where f=ω/2π.

From Figure 3, Figure 4, Figure 5 and Figure 6, it can be seen that as the excitation frequency increases, the equivalent impedance across the electrode plates tends to stabilize, and the impact of unknown capacitance in the equivalent circuit on the entire system is small, with the equivalent impedance across the electrode plates approximately equal to the solution resistance. The analysis of multiple frequency ranges provides a complete understanding of the system’s frequency response characteristics. The above simulation results point the direction for our subsequent selection of experimental conditions. Therefore, experiments were only further calibrated in the 1–3 MHz frequency band to obtain optimal measurement accuracy.

**Figure 7:**
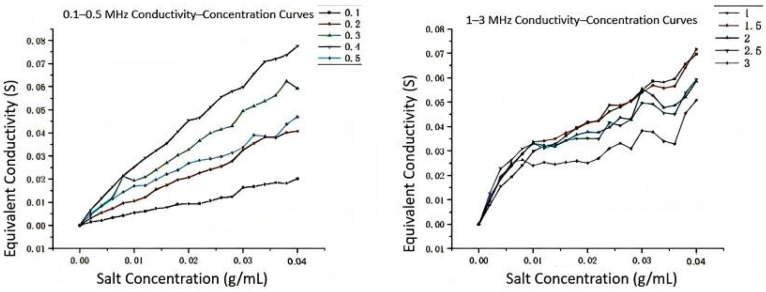
0.1–0.5 MHz and 1–3 MHz Conductivity–Concentration Curves.

**Figure 8:**
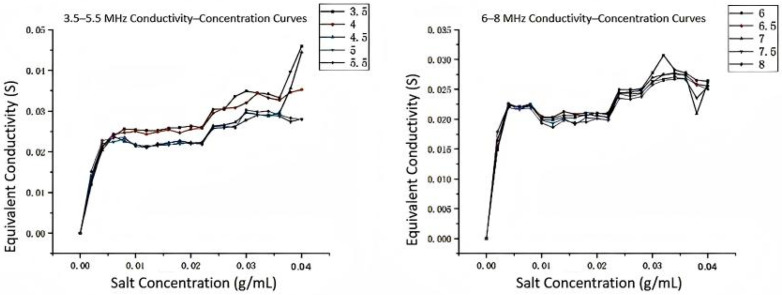
3.5–5.5 MHz and 6–8 MHz Conductivity–Concentration Curves.

The experimental results showing the conductivity–concentration relationships at different frequency ranges are presented in Figure 7 and Figure 8. These curves demonstrate the frequency-dependent behavior of the measurement system and validate the theoretical analysis.

### 3.2. Applicable Prerequisites of Equivalent Circuit

In this experiment, the excitation frequency involved in the circuit is high. Under high-frequency excitation, the electromagnetic field changes in the circuit are complex, and Kirchhoff’s first law and Kirchhoff’s second law are no longer applicable, i.e., the equivalent circuit no longer satisfies the quasi-steady condition and lumped condition [17]. Therefore, when selecting equivalent circuit external excitation conditions, the magnitude of the excitation frequency is a key factor that needs to be carefully considered and examined: first, when the excitation frequency is too low, the impedance of the double-layer capacitance cannot be ignored, it is not satisfying the circuit simplification conditions, which means the double-layer capacitance has a large impact on the experimental circuit, not satisfying the lumped condition of the circuit; second, when selecting too high frequency, the spatial wavelength is short, not satisfying the linearity condition l≪λ (l is the circuit size), and the quasi-steady condition does not hold. According to field measurements, the circuit size in this experiment is l≈1 m. Estimating with frequency 0.1–20 MHz, the wavelength λ is about 15–3000 m, which is much larger than the circuit size, so the quasi-static approximation condition is basically satisfied in the experimental frequency band.

At this time, the current I flowing through the solution to be measured is approximately equal to the current flowing through the voltage divider resistance $R_{1}$, and the conductivity G of the solution to be measured (the reciprocal of resistance R) can be calculated by Equation (6):

(6) $$G=\frac{1}{R}=\frac{I}{U_{p}}\approx \frac{U_{1}/R_{1}}{U_{p}}.$$

where R is the real part of $Z_{p}$, approximately equal to the pure resistance of the solution under high-frequency excitation.

This simplified model is the basic principle for subsequent experimental measurements.

## 4. Device Design and Experimental Model Fabrication

### 4.1. Overall Design

**Figure 9:**
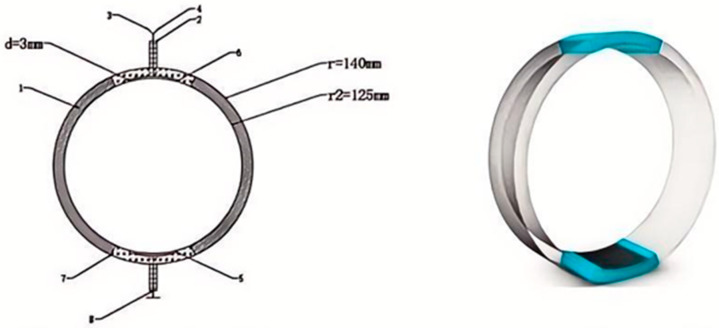
Schematic and 3D Model of the In-Pipeline Curved-Electrode Conductivity Sensor.

Based on field research results of shipboard seawater desalination systems, combined with pipeline structure and parameters, we designed the measurement device as shown in Figure 9. This device can be connected to the original transmission pipeline through flanges and measure its concentration in real-time when the seawater to be measured flows through.

Table 1 in the figure is the glass steel pipe wall, with inner diameter of 125 mm, outer diameter of 140 mm, and length of 200 mm; 2 is the external protective layer of insulated cable, made of insulating rubber; 3 and 4 are two insulated wires, respectively, connected to the excitation source and output sampling signal; 5 is the lower platinum electrode plate, with central angle of 30°, thickness of 3 mm, centered and combined with the pipeline, length of 100 mm; 6 is the upper platinum electrode plate, with central angle of 30°, thickness of 3 mm; 7 is the electrode protective insulating layer, made of glass steel; and 8 is the grounding wire.

**Figure 10:**
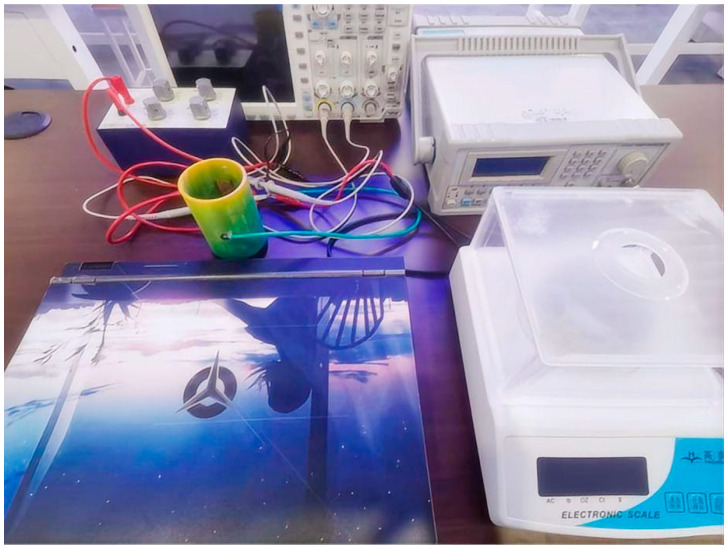
Experimental Setup for In-Pipe Conductivity Measurement System.

After applying excitation electrical signals to the electrode plates, the electrode voltage and circuit current are sampled and converted through A/D conversion and input to the computer, which can quickly process and obtain the equivalent conductivity of the solution and the corresponding solution concentration, while also transmitting data to other control devices, achieving linkage control in multiple fields. The complete system setup is shown in Figure 10.

### 4.2. Experimental Model Fabrication

**Figure 11:**
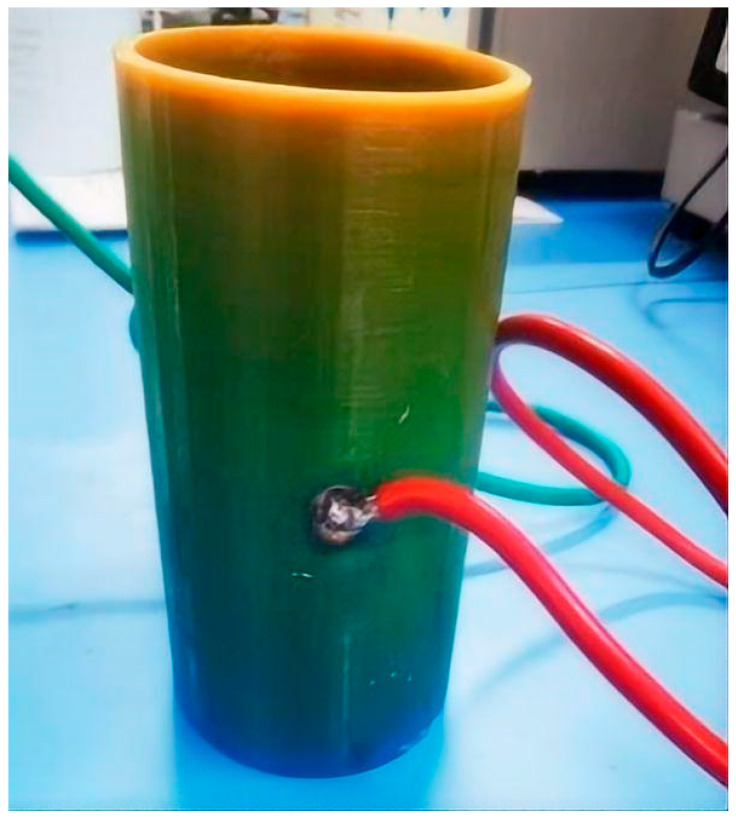
Photograph of the 3D-Printed Pipeline with Embedded Curved Electrode.

**Figure 12:**
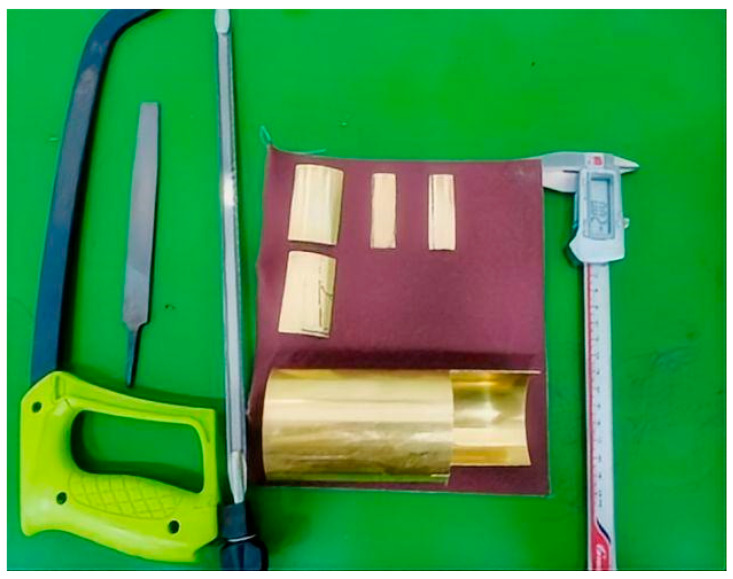
Fabrication of the Copper Electrode Plates.

For convenient laboratory performance testing, we used 3D printing technology to fabricate experimental models. The pipeline was 3D printed using nylon material, with design specifications of inner diameter 64 mm, outer diameter 68 mm, and wall thickness 2 mm. Grooves were preset on the inner wall of the pipeline, with length of 100 mm, for embedding cylindrical electrode plates. The physical realization of this design is shown in Figure 11. Copper sheets were used instead of platinum for the electrode plates, processed into curved shapes with the same diameter as the pipeline inner diameter using manual saws, files, sandpaper, and other tools, with length of 50 mm and width consistent with the line width of the preset grooves on the pipeline inner wall. The fabrication process of the custom-designed copper electrode plates is illustrated in Figure 12.

The electrode plates were securely mounted in the pipeline grooves using adhesive, with proper insulation and waterproofing treatment. Electrical connections were made through soldering, and the assembly was sealed with UV-cured resin to ensure reliable operation.

### 4.3. Test Circuit Design

The test circuit was designed according to the equivalent circuit model. A digital signal generator provided excitation signals, while a dual-channel oscilloscope sampled the electrode voltage U and divider voltage $U_{1}$. The voltage divider resistance $R_{1}$ was selected to match the solution resistance magnitude for optimal measurement accuracy.

For each concentration–frequency combination, 20 repeated measurements were performed and averaged. This testing protocol generated 18,000 data points across 20 concentration levels and 45 frequency points.

## 5. Device Performance Testing

### 5.1. Impact of Excitation Frequency on Measurement

To determine the optimal working frequency band of the online detection device, we conducted frequency sweep experiments. Sodium chloride was gradually added to 250 mL of distilled water to prepare 20 solutions with concentrations from 0.2% to 4.0%. For each concentration solution, excitation signals with amplitude of 10 V and frequency varying from 0.1 MHz to 20 MHz were applied, recording the values of $U_{p}$ and $U_{1}$, and calculating the equivalent conductivity G of the solution.

All data were plotted as conductivity–frequency relationship curves. Analysis results show the following:

- In the low-frequency band (<1 MHz), conductivity values change dramatically, with frequency and curves of different concentrations crossing, with poor linearity, unsuitable as a working area.
- In the high-frequency band (>6 MHz), although curves tend to flatten, the distinction between curves of different concentrations decreases, and high-frequency noise interference increases.
- In the **1–3 MHz** frequency band, curves of various concentrations show good consistency and distinction, with relatively stable conductivity values. This frequency band was selected as the **optimal working frequency band** of the device, determining key parameters for subsequent precise measurements.

The conductivity–concentration linearity comparison at different frequencies shows that the 1.5 MHz frequency provides the best linear relationship, making it ideal for accurate concentration measurements.

### 5.2. Calibration of Device Working Curve

**Figure 13:**
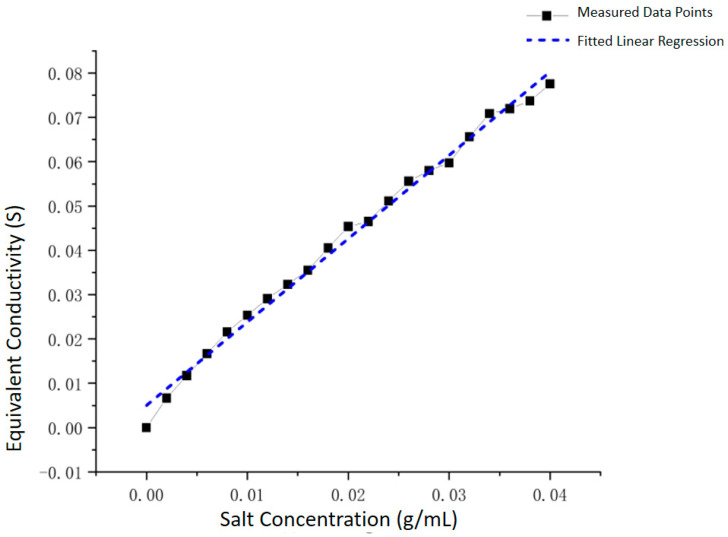
Calibration Characteristic of the Conductivity Measurement Device.

Selecting the working frequency as **1.5 MHz**, we measured the relationship between solution conductivity value G and salt content c (g/mL) in the concentration range from 0.2% to 4.0%. Linear fitting was performed on the data points to obtain the working curve of the online detection device, as shown in Figure 13.

The fitting results show that the two have high linear correlation, with **correlation coefficient R^2^ reaching 0.99319**. The detailed fitting parameters are shown in Table 2. The fitted straight-line equation is as follows:

(7) $$c\;\left(g/mL\right)=\frac{G\;\left(S\right)-0.005}{1.88242}$$

This equation is the calibration equation of this measurement device, which can directly convert solution concentration c by measuring conductivity value G, providing an accurate mathematical model for the engineering application of the device.

### 5.3. Device Effectiveness Verification

To verify the accuracy of the online detection device, we used the calibrated device to measure two standard solutions:

- **Solution A**: Commercially available physiological saline (standard concentration ≈ 0.9%).
- **Solution B**: Laboratory-prepared simulated seawater (standard concentration = 3.5%).

Experimental results show that our designed device can accurately measure salt solution concentration, with higher accuracy, especially at low concentrations. The measurement error for simulated seawater is slightly higher, mainly due to systematic errors in laboratory preparation and measurement processes. These results verify the effectiveness of the device design and lay an experimental foundation for subsequent engineering applications. Further experimental data are provided in the Supplementary Materials.

### 5.4. Uncertainty Analysis

For each solution, we performed five repeated measurements of the voltage $U_{i}$ and computed the Type–A uncertainty:

(8) $$\left(U\right)=\sqrt{\frac{\sum_{i=1}^{5}(U_{i}-\bar{U}{)}^{2}}{5-\left(5-1\right)}}\;.$$

The Type–B uncertainty due to the voltage divider resistor error Δm is as follows:

(9) $$U_{B}\left(U_{1}\right)\;=\;U_{B2}\left(U_{2}\right)\;=\;\frac{\Delta m}{\sqrt{3}}\;.$$

Propagating these through the calibration function $G\left(U\right)$ gives the combined standard uncertainty as follows:

(10) $$U_{G}=\sqrt{(\frac{\partial G}{\partial u_{1}}\;U_{A}(u_{1}){)}^{2}+(\frac{\partial G}{\partial u_{2}}\;U_{B2}(u_{2}){)}^{2}}\;.$$

Using the measured $U_{i}$ arrays and Δm, we obtain for Solution A and B:

(11) $$G_{A}=0.0225\pm 0.0005\;S,\;G_{B}=0.0718\pm 0.0022\;S,$$

Which correspond to the following concentrations:

(12) $$c_{A}=9.167\pm 0.056\;g/L,\;c_{B}=3.551\pm 0.226\;g/L.$$

### 5.5. Error Source and Application Range Analysis

Through experimental analysis, we identified the following main error sources:

1. **Temperature effects**: The experiment was not strictly temperature-controlled. According to relevant data, for salt solutions with concentration higher than 1.3%, their conductivity is greatly affected by temperature. Although the laboratory temperature was controlled around 25.5 °C, there were still temperature variations affecting experimental results.
2. **Concentration nonlinearity**: Experimental data show that the device has higher measurement accuracy for low-concentration salt solutions than high-concentration salt solutions, especially when concentration is below 1%, showing higher precision. This error is primarily due to the increased inter-ionic interactions at higher concentrations, which non-linearly reduce ion mobility and complicate the conductivity–concentration relationship, as explained above.

This observed higher accuracy at lower concentrations can be attributed to several fundamental factors:

a. Ion-Ion Interactions: At lower concentrations, the average distance between ions is greater, which minimizes the electrostatic interactions between them (such as the relaxation and electrophoretic effects). This results in a more linear and predictable relationship between conductivity and concentration, making measurements more accurate [10]. As the concentration increases, these inter-ionic forces become significant, leading to non-linear deviations from ideal behavior and thus increased measurement uncertainty.

b. Signal-to-Noise Ratio (SNR) Advantage: “The measurement system itself, including the electronics, has a inherent noise floor. At lower concentrations, the relative change in impedance (or conductance) per unit change in concentration is more significant compared to this noise floor, yielding a higher signal-to-noise ratio and, consequently, better precision.”

c. Double-Layer Effects: “For the two-electrode system employed, the electrical double layer (EDL) at the electrode-solution interface has a more stable and less disruptive influence in dilute solutions. In highly concentrated solutions, the compressed thickness of the EDL can lead to more complex impedance characteristics, potentially introducing errors.”

3. **Electrochemical reactions**: Complex, difficult-to-measure electrochemical reactions exist between the energized electrode plates and the solution in the experiment. Although the selection of working points minimizes errors caused by such reactions as much as possible, because the intensity of electrochemical reactions is uncontrollable and random, there are still non-negligible errors.

**Application range**: The literature review shows that since inorganic salt ions in salt solutions begin to ionize in large quantities when concentration >5%, their conductivity rise is not linear, requiring correction using the Debye–Hückel limiting formula. Therefore, the experimental conclusions and methods are applicable to solutions with concentration <5%, but not suitable for measuring salt solutions with higher concentrations. However, since seawater concentration is generally around 3.5% and desalinated seawater concentration is below 3.5%, this device can still meet the measurement requirements of salt concentration in the seawater desalination process.

A comparison with state-of-the-art conductivity-sensing methodologies highlights the distinct advantages of our conformal curved-electrode design. Conventional systems, often reliant on parallel-plate or multi-electrode configurations, face inherent challenges for in-pipeline integration. For instance, systems based on variable-frequency excitation of traditional electrodes [18] must contend with signal instability caused by unpredictable air gaps (d_g) when mounted on curved surfaces. While highly precise multi-electrode cells (e.g., 7-electrode systems [19]) exist for laboratory-grade accuracy, their structural complexity and reliance on sophisticated board-level temperature compensation algorithms present barriers for robust, low-cost industrial deployment. Recent advancements in electrode optimization, even those leveraging AI-based modeling [20], often remain focused on the material properties within a conventional form factor.

In contrast, our sensor addresses the fundamental issue of geometry and integration. By eliminating the air gap through a perfectly conformal curved design, we achieve a stable and predictable measurement interface. The performance of this structural innovation is validated through extensive testing. The sensor demonstrates a wide operating range, with effective calibration curves established across the entire range of 0.1% to 4.0% concentration. Crucially, it achieves high accuracy in real-world media: the relative error after calibration is 3.000% for commercially available medical saline and 1.457% for simulated seawater (3.5% NaCl solution). This robust performance, achieved directly within pipeline systems, obviates the need for the complex compensation circuitry required by bulkier designs and provides a more streamlined and practical solution for real-time seawater concentration monitoring.

### 5.6. PLC Control System Integration

To enhance the practicality of the online detection device, we further attempted to integrate it with a Programmable Logic Controller (PLC) control system. Through an advanced signal processing circuit, high-frequency voltage signals were converted to DC signals that can be collected by PLC, and ladder logic was written to achieve automatic data conversion and result display, realizing real-time online monitoring of seawater concentration in pipelines.

We selected Xinjie XD3−16R and XD-E4AD voltage expansion module as the PLC module for the experimental device, and selected Xinjie TG765S-XT touch screen as the display and control terminal. Since PLC has difficulty sampling high-frequency signals, the voltage signal was converted to DC signal through a single-phase bridge rectifier and then connected to the analog input terminal of the PLC expansion module. The device voltage was connected to CH0 channel, stored by D0 register; the load voltage was connected to CH2 channel, stored by D2 register.

Through practice, it was found that adding PLC devices does not affect test results; data processing and display are very convenient and fast, improving test efficiency, making the device more streamlined, fitting actual scenarios, and more conducive to practical applications in shipboard desalination systems. Using the experimental device, with PLC module to measure commercially available physiological saline, the display interface showed results of G = 22 mS and C = 9 g/L (about 0.9% concentration, consistent with Equation (7) calculation results), verifying the reliability of the system.

## 6. Conclusions

This study developed an online conductivity measurement system for shipboard seawater desalination. The work addresses key limitations in current maritime water treatment monitoring through curved electrode design and systematic optimization.

### 6.1. Technical Achievements

The study developed and tested a new measurement system with the following main results:

1. **Curved Electrode Design**: Changed flat electrodes into curved ones that fit inside pipelines. This design reduces flow disturbance while keeping measurement accuracy. The electrodes can be installed in existing pipelines without major changes.
2. **Theoretical Model**: Built an equivalent circuit model for high-frequency measurements. The model includes electrode polarization, double-layer capacitance, and wire effects. This provides a basis for system design.
3. **Frequency Analysis**: Used MATLAB to study frequency response from 0.1 to 20 MHz. Found the best working frequency is 1–3 MHz, with 1.5 MHz giving the best linear fit (R^2^ = 0.99319) between conductivity and concentration.
4. **Experimental Testing**: Made 18,000 measurements across 20 concentration levels and 45 frequency points. Error analysis showed 0.10% error for low-concentration solutions and 2.20% for high-concentration solutions.
5. **PLC Integration**: Connected the system to a PLC for automatic measurement and display. This allows continuous monitoring without manual work, improving efficiency in real ship environments.

### 6.2. Scientific Contributions

The research contributes to conductivity measurement and maritime water treatment in the following ways:

1. **New Method**: Combined theory, simulation, and experiments for conductivity measurement. This approach works reliably under different conditions.
2. **Parameter Optimization**: Found the best working parameters through frequency analysis. The 1.5 MHz frequency gives better accuracy than traditional methods and reduces electrochemical interference.
3. **Practical Application**: Showed that the system works in real ship environments. It handles ship vibration, temperature changes, and harsh conditions, making it suitable for widespread use.
4. **Data Analysis**: Used 18,000 measurements to build calibration curves and error analysis. This ensures reliable performance across the 0.2% to 4.0% concentration range.

### 6.3. Practical Implications

The system solves key problems in maritime water treatment:

1. **Real-time Monitoring**: Provides continuous concentration monitoring during desalination, giving immediate feedback on system performance and water quality.
2. **Distributed Deployment**: Can be installed at multiple points in the desalination system, covering the entire treatment process.
3. **Operational Efficiency**: Reduces maintenance needs and improves system reliability through automated monitoring and early problem detection.
4. **Cost Effectiveness**: Reduces operational costs by preventing unnecessary filter replacement and optimizing system performance through continuous monitoring.

### 6.4. Future Research Directions

The study suggests the following areas for future work:

1. **Extended Concentration Range**: Study measurement capabilities for concentrations above 5%, which may need modified electrode designs and measurement methods.
2. **Temperature Compensation**: Develop temperature compensation methods to improve measurement accuracy under changing environmental conditions.
3. **Multi-Parameter Sensing**: Add other water quality parameters (pH, turbidity, and dissolved oxygen) to create a comprehensive monitoring system.
4. **Advanced Signal Processing**: Use machine learning for better data analysis and predictive maintenance.

### 6.5. Conclusions

This research shows that curved electrode conductivity measurement works well for shipboard seawater desalination systems. The combination of theory, simulation, and experiments produced a reliable measurement system with good accuracy and practical use. The system’s real-time, distributed monitoring capability represents an important step forward in maritime water treatment technology, improving operational efficiency and system reliability.

The technology has been tested extensively and is ready for practical use in maritime environments. The PLC integration makes the system more useful and suitable for widespread use in shipboard desalination systems worldwide.

## Figures and Tables

**Table 1:** Linear Fit Parameters.

Field	Value
Equation	y=a+b x
Plotted Quantity	Equivalent Conductivity
Weighting	Unweighted
Intercept	$0.005\pm 8.36\times 10^{-4}$
Slope	1.88242±0.03576
Sum of Squared Residuals	$7.48198\times 10^{-5}$
Pearson’s r	0.99659
R-squared (COD)	0.99319
Adjusted R-squared	0.99283

**Table 2:** Test results of liquid concentration to be measured.

Solution Type	Measured Conductivity G (S)	Converted Concentration c (%)	Actual Concentration c (%)	Relative Error
Solution A	0.022	0.92	0.91	0.10%
Solution B	0.070	3.55	3.63	2.20%

## Data Availability

Data supporting the results of this study are available from the corresponding author upon reasonable request.

## Supplementary Materials

The following supporting information can be downloaded at: https://www.mdpi.com/article/10.3390/s25175464/s1.

## References

[1] Sun B.Q., Zhang X.L., Xing D.Y., Li J., Dong W.Y., Pan X.H. (2021). Development and application of seawater desalination technology. Guangdong Chem. Ind..

[2] Wang H.B. (2011). Research on pH and conductivity online detection system for seawater desalination. Master’s Thesis.

[3] Pan L., Zhu Q.Y., Li Y., Huang S.S., Song D. (2016). Research on copper ion concentration monitoring system based on conductivity electrode. Tech. Exch..

[4] Liu H.B., Zhao L.Z., Zhu P.Q., Shi Y.T., Peng A.W. (2022). Optimal design of magnetohydrodynamic wave generator under real sea conditions. Acta Energiae Solaris Sin..

[5] Pang L., Dong H., Zhao X., Feng J., Fang L., Zhao N., Zhang Z. (2023). Frequency adaptive conductivity measurement based on reverse approximation. Measurement.

[6] Freye C., Jenau F. (2018). Model-based accuracy enhancements for guarded conductivity: Determination of effective electrode areas utilising numerical field simulation. High Volt..

[7] Rustomji C.S., Mac J., Choi C., Kim T.K., Choi D., Meng Y.S., Jin S. (2015). Thin-film electrochemical sensor electrode for rapid evaluation of electrolytic conductivity, cyclic voltammetry, and temperature measurements. J. Appl. Electrochem..

[8] Liu J., Zhang Z., Wang Y., Zheng J., Guo Y., Yao B., Zhang S., Jing J., Xu Y., Xue C. (2025). A board-level temperature compensation method for precise seawater conductivity measurement. Sens. Actuators A Phys..

[9] Chen L.M., Cheng M.X., Xiao X.F., Huang Z.H. (2010). Measurement of the relationship between conductivity and concentration and temperature of salt solution. Lab. Res. Explor..

[10] Fu X.C. (2009). Physical Chemistry (Volume 2).

[11] Dong W.F., Huang Z.Q. (2017). Determination of EDTA disodium salt solution concentration by conductivity method. Guangzhou Chem. Ind..

[12] Lin Z., Zhang X., Wei J.L., Wang X.P. (2015). Solution conductivity meter based on Van Der Pauw principle. J. Zhejiang Univ. (Eng. Sci.).

[13] Jia Z.L., Zhao C., Leng X.J. (2015). Research on a dual-frequency conductivity measurement method. Autom. Technol. Appl..

[14] Lu J.B. (2019). Measurement principle and application of WDD-IV conductivity sulfuric acid concentration analyzer. Sulfuric Acid Ind..

[15] Wang X.J. (2018). Circuit Theory.

[16] Ni G.Z. (2016). Engineering Electromagnetic Field Theory.

[17] Zhang J.X. (2010). Analysis of the relationship between quasi-steady conditions, lumped conditions and circuit equations. Gansu Sci. Technol..

[18] Hu S., Wu K., Wang H., Chen J. Electrical conductivity measurement method in seawater desalination based on variable frequency excitation. Proceedings of the 2009 9th international Conference on Electronic Measurement & Instruments.

[19] Mazzeo B.A., Flewitt A.J. (2007). Two- and four-electrode, wide-bandwidth, dielectric spectrometer for conductive liquids: Theory, limitations, and experiment. J. Appl. Phys..

[20] Khalil A., AlShabi M.A., Abdelrazek K., Obaideen K. Optimization of capacitive deionization electrode features and materials using artificial intelligence-based modeling. Proceedings of the Energy Harvesting and Storage: Materials, Devices, and Applications XIV.

