# Supplementary material for: Conformal Curved-Electrode Sensor with High-Frequency Optimization for Distributed Conductivity Monitoring in Shipboard Desalination Pipelines
Source: Sensors (Basel). 2025 Sep 3;25(17):5464. doi: 10.3390/s25175464 (PMC12431524; doi:10.3390/s25175464)
Supplement: Supplementary file 1 [file sensors-25-05464-s001.zip › Supplementary Data File 1 (raw_data.xlsx)/Chinese-English Translation Guide.pdf]

In accordance with the request to translate all text within the supplementary materials into English, please find below a comprehensive translation guide for the column headers and parameters used in the attached data tables. This guide ensures clarity and accurate interpretation of all data presented.

Data Table Translation Guide:

中文 **(Chinese)**	**English Translation**	**Notes / Explanation**
频率	**Frequency**	Measurement frequency of the electrical signal.
距离	**Distance**	Distance between measurement points (in meters, m).
电导池常数	**Cell Constant**	Denoted as **k**, a geometric factor of the conductivity cell.
万用表 **U1**	**Multimeter U1**	Voltage reading (in Volts, V) from multimeter 1.
定值电阻	**Fixed Resistor**	Resistance value (in Ohms, Ω) of the reference resistor.
初始读数	**Initial Reading**	Initial value before measurement (context-dependent).
电流	**Current**	Electrical current (in Amperes, A), calculated as I = U1 / R.
示波器 **VPP**	**Oscilloscope V_{PP}**	Peak-to-peak voltage (in Volts, V) measured by the oscilloscope.
幅值	**Amplitude (V_m)**	Peak voltage amplitude (in Volts, V), half of V_{PP}.
有效值	**RMS Value**	Root Mean Square voltage (in Volts, V), calculated as V_m/√2.

中文<br>**(Chinese)**	**English Translation**	**Notes / Explanation**
电流均值	**Mean Current**	Average value of the current measurements.
均值	**Mean Value**	Average value of the RMS voltage measurements.
电导率	**Conductivity**	Electrical conductivity of the solution (in S/m or S/cm).
